# Editorial: Biological rhythms in the brain and gastrointestinal tract

**DOI:** 10.3389/fphys.2025.1622475

**Published:** 2025-05-15

**Authors:** Jingling Jin, Nange Jin, Byung Joo Kim, Maria Nathalia Moraes, Leonardo V. M. de Assis

**Affiliations:** ^1^ University of Texas MD Anderson Cancer Center, Houston, TX, United States; ^2^ Department of Vision Sciences, University of Houston College of Optometry, Houston, TX, United States; ^3^ Department of Longevity and Biofunctional Medicine, Pusan National University School of Korean Medicine, Busan, Republic of Korea; ^4^ Departamento de Ciências Biológicas, Instituto de Ciências Ambientais, Químicas e Farmacêuticas, Universidade Federal de São Paulo, São Paulo, Brazil; ^5^ Department of Chemistry and Molecular Biology, University of Gothenburg, Gothenburg, Sweden; ^6^ Wallenberg Centre for Molecular and Translational Medicine, University of Gothenburg, Gothenburg, Sweden

**Keywords:** biological rhythm, circadian, entrainment, metabolism, ultradian, cancer, gastrointestinal tract, visual system

Biological rhythms, especially circadian (∼24-hour) oscillations, orchestrate essential physiological processes across multiple organs. The central circadian clock, located in the suprachiasmatic nucleus (SCN) of the hypothalamus, generates endogenous rhythms and temporally coordinates peripheral clocks in organs, including those of the gastrointestinal (GI) tract. This synchronization aligns internal physiology with external environmental cues (zeitgebers), such as light-dark cycles and feeding times, as well as internal signals influenced by them, including microbiota-derived factors.

At the cellular level, circadian rhythms, in central and peripheral clocks, are driven by transcriptional–translational feedback loops involving core clock proteins, including CLOCK and BMAL1, which activate the expression of *Pe*r and *Cry*; these, in turn, inhibit their own transcription, generating a ∼24-hour cycle of gene expression. This integrated clock network regulates behavior, metabolism, hormone secretion, digestion, and immune responses. Disruptions to these rhythms—due to irregular light exposure, shift work, or altered meal timing—can desynchronize central and peripheral clocks, increasing vulnerability to disorders such as metabolic syndrome, cancer, gastrointestinal diseases, and mood disorders (Panda, 2016; Voigt et al., 2014; Sulli et al., 2018). The present Research Topic synthesizes current knowledge on the mechanisms of circadian rhythms, their susceptibility to disruption, and their implications for health and disease.

One core focus of this Research Topic is the role of the gut microbiome in circadian biology. Emerging research suggests that the gut microbiota exhibits circadian fluctuations, influencing host rhythms through microbial metabolites. However, Ehichioya et al. indicate that circadian behavioral rhythms may be resilient to changes in microbiota composition. The experimental depletion of gut microbiota minimally affects circadian locomotor and food-anticipatory activities in mice, suggesting strong central-clock dominance. This contrasts with previous findings of microbiota-driven circadian alterations (Thaiss et al., 2016; Brooks et al., 2021), underscoring the complexity and context-dependent nature of microbial-host clock interactions.

Hormonal modulation significantly impacts peripheral clocks. Thyroid hormones, crucial regulators of metabolism, also synchronize intestinal clocks, affecting rhythmic nutrient absorption. The findings from Secio-Silva et al. show that hypothyroidism in female mice disrupts circadian gene expression in the gut, eliminating rhythmic fluctuations of key nutrient transporters involved in lipid and carbohydrate absorption. Importantly, thyroid hormone effects are tissue-dependent, as either low or high thyroid hormone levels have a small effect on the expression of core clock genes in the liver (de Assis et al., 2022; de Assis et al., 2024).

Circadian rhythms prominently govern gastrointestinal physiology. Colonic functions—including motility, nutrient absorption, hormone secretion, and mucosal integrity—exhibit distinct circadian rhythmicity, regulated by clocks within the enteric nervous system and intestinal epithelial cells. Such important concepts and knowledge were elegantly summarized by Hibberd et al. Interestingly, the gut synthesizes substantial levels of melatonin, a classical circadian hormone primarily known for its secretion by the pineal gland. Melatonin locally modulates gut inflammation and motility, highlighting its potential therapeutic role for GI disorders exacerbated by circadian misalignment (Zimmermann et al., 2024).

Chronodisruption—circadian misalignment due to inappropriate exposure to environmental zeitgebers—has profound health implications throughout the lifespan. The findings from González-González et al. demonstrate that maternal exposure to dim light at night (DLAN) simulates chronic circadian disruption and induces persistent behavioral and neural alterations in offspring. Notably, male offspring of DLAN-exposed mothers exhibit reduced social behaviors, increased anxiety-like behaviors, and enhanced microglial activation in brain regions associated with reward and social interaction (Mendez et al., 2016; Mendez et al., 2022; Halabi et al., 2021). These findings show how prenatal circadian disruption can impose lasting developmental consequences, potentially mediated through neuroimmune pathways. Such results align with evidence linking prenatal circadian stressors to neurodevelopmental and psychiatric disorders in mice (Delorme et al., 2025).

Circadian misalignment caused by occupational factors, especially night-shift work, presents a significant epidemiological concern. Chronic shift work exposure is classified by the International Agency for Research on Cancer (IARC) as a risk factor for human carcinogen (Straif et al., 2007). Epidemiological studies indicate elevated risks for gastrointestinal cancers, particularly colorectal cancer, among long-term night-shift workers. Although findings vary, reflecting complexities in study designs, occupational settings, and individual susceptibility, accumulating evidence strongly implicates disrupted circadian regulation in tumorigenesis. The review by Guo and Li elegantly addresses this complex regulation. Proposed mechanisms include impaired DNA repair, altered metabolism, chronic inflammation, and suppressed nocturnal melatonin secretion, each linking circadian disruption directly to increased cancer vulnerability (Straif et al., 2007; Schernhammer et al., 2003). Continued epidemiological and mechanistic research is essential for refining preventive strategies, especially in occupations involving chronic circadian disruption.

While circadian rhythms dominate biological timing research, recent findings identify ultradian (approximately 12-hour) rhythms as critical regulators of physiology. Distinct from the SCN-driven circadian clock, these ultradian oscillations modulate processes such as protein homeostasis, stress response, and metabolism, largely through cell-autonomous mechanisms involving transcription factors like XBP1 (Meng et al., 2020). Such rhythms, particularly evident in the liver and potentially in mammary tissue, highlight additional layers of temporal regulation that are essential for maintaining cellular and systemic homeostasis. The review from Dion and Zhu provides an insightful view on recent evidence for ultradian rhythms. Investigating these ultradian rhythms opens new avenues for translational research and therapeutic timing interventions, particularly relevant for metabolic and proteostatic disorders.

Collectively, contributions to this Research Topic illustrate the intricate interplay of biological rhythms across neuroscience, gastroenterology, endocrinology, immunology, and epidemiology. These studies emphasize the necessity of maintaining internal rhythmic alignment to optimize health and mitigate disease risks. They further advocate for integrative chronobiological approaches in clinical practice—leveraging meal timing, controlled light exposure, melatonin therapy, and scheduled drug administration—to restore rhythmic integrity disrupted by modern lifestyles. Future directions must include refining methodologies to assess circadian disruption in real-world settings, exploring sex-specific vulnerabilities to chronodisruption, and developing chronotherapeutic strategies tailored to individual circadian profiles. Enhancing interdisciplinary research on brain-gut rhythms promises substantial advancements in preventive medicine and personalized healthcare.

In conclusion, maintaining synchronized biological rhythms in the brain and gastrointestinal functions is essential for health. Interdisciplinary efforts in physiology, chronobiology, neuroscience, and gastroenterology will continue to uncover how precise timing interventions can prevent and treat disorders linked to circadian disruption, ultimately promoting better human health in an increasingly time-challenged society.
